# Cases Illustrative of the Curative Effects of Copious and Long-Continued Affusions of Cold Water in External Inflammations

**Published:** 1828-08

**Authors:** L. S. Tillet

**Affiliations:** Lancaster, Pennsylvania.


### COLD WATER.

*Cases illustrative of the Curative Effects of copious and long-continued Affusions of Cold Water in external Inflammations.* By L. S. Tillet, m.d. of Lancaster, Pennsylvania.*

In the early part of his life, Dr. —— had been afflicted with a white swelling of the knee-joint, from which he had the good fortune to escape with an anchylosis; which, while it occasioned him some little inconvenience, in no degree incapacitated him for a faithful discharge of his professional duties, during a period of more than twenty years. In descending the steps of his house, some time since, he unfortunately slipped, and the heel of the anchylosed limb meeting with the projecting point of a stone, which was firmly fixed in the earth, a fracture took place at the knee-joint, and the leg was doubled beneath him. With the assistance of a friend, the parts were replaced as nearly as possible in their original situation; and, having been removed into the house, medical assistance was immediately procured: it was to little purpose, however, as, notwithstanding the excruciating pain which he suffered, he had already determined on the treatment to be pursued. Convinced, by long experience, of the utility of cold applications in cases of this nature, he resolved to confide his life to them alone; and, throughout his whole confinement, pertinaciously refused to submit to venesection, or to take purgative medicine in sufficient quantity to do more than maintain the regular peristaltic action of the intestines. Indeed, his great size, and the exquisite pain attendant upon the least motion, limited his friends to the exhibition of mild laxatives only. By this time the knee had become much swollen, and it was evident that whatever plan was adopted must be vigorously pursued. The leg was extended upon a pillow, no dressings of any kind applied, and the coldest water that could be procured was poured upon the naked limb, at first almost constantly, and afterwards, as the inflammation subsided, in quantity of a pint every five and ten minutes, for three weeks. At the expiration of this period, having been removed to a dry bed, and almost exhausted by the continued state of submersion in which he had been for so long a time, he resolved to try the effect of cloths moderately wet with a solution of the acetate of lead,

* Condensed from the North American Med. and Surg. Journal.

&c. In less than three hours, a most marked difference had taken place in the state of the limb: the inflammation had greatly increased; the pain had become almost insupportable, shooting upwards to the body, and downwards to the foot. A vesicle, five lines in diameter, containing a yellowish serum, made its appearance on the superior part of the joint; and every symptom, in short, bore evidence of the impending danger. No time was to be lost: cold water was instantly had recourse to, in quantity as great as in the first instance, and with the same salutary consequences. The inflammation again subsided, with a consequent diminution of pain; the vesicle became shrivelled, from an absorption of its contents, and ultimately disappeared altogether. A continuance of this plan, proportioned to the degree of inflammation, for three weeks longer, effected a complete cure; and, in eight weeks from the occurrence of the accident, he was able to walk with the assistance of crutches.

A more unequivocal proof of the powerful agency of cold water than this case affords, cannot be desired. The patient was extremely corpulent, weighing upwards of three hundred pounds. The inflammatory action ran so high, that the water would rise from the limb in vapour. The accident occurred in the month of August; and, with the exception of a very abstemious diet, there was no other remedy employed. It may be said, perhaps, that general and local depletion, vigorously pursued, would have been attended with a like beneficial result; but to the former the prejudices of the patient opposed an insuperable objection; and besides that, in the country, and at a distance from any considerable town, (as was the case in this instance,) leeches cannot always be procured, there is great reason to think that they would have been totally inadequate to subdue the violent inflammation which existed here, and which, upon a mere relaxation of the treatment at the expiration of three weeks, threatened the life of the patient.

Another instance, in which the same remedy was successfully employed, communicated to me by the gentleman whose case I have just related, is little less remarkable, inasmuch as it is a case for which, not many years ago, amputation would have been deemed necessary.

A woman, jumping out of a waggon, the horses of which were unruly, dislocated the tibia at the ankle; the bone protruded through the integuments, and penetrated the earth to some distance. The dislocation was reduced by a surgeon who happened to be near at the time; and the day following, the woman having been conveyed to her house, Dr. —— was sent for. He found the leg much swollen and inflamed, and the patient in great agony. Cold water was instantly applied, and with the most salutary consequences. In five weeks the patient was able to walk about the house; though, from the nature of the injury, the joint was affected for some time after with a considerable degree of stiffness.

I could adduce many instances of compound fractures, in which the free employment of this remedy was attended with a like happy result. Among others, one which occurred in my own practice, in which both the bones of the leg were fractured, and the integuments much lacerated, the patient being at the time in a state of inebriety. The inflammation was restrained within due limits, and although, from the injury done to the integuments, union by the first intention did not take place, the cure progressed without a single unpleasant symptom.

But enough has surely been said to prove that cold, *properly* applied, is possessed of much higher remedial power than has hitherto been attributed to it. I say properly, because the usual manner in which it is employed, by means of cloths wet with a solution of acetate of lead and other substances, removed as often as they become dry by evaporation, is productive of comparatively little advantage: it may be useful in mild cases, but in the more violent grades of inflammation, must be altogether nugatory. Here a more vigorous treatment is demanded: the coldest spring or well water should be procured, or, if necessary, it may be cooled artificially, and the limb be kept constantly bathed in it, until the morbid excitement be reduced; and this, I hesitate not to say, in a constitution otherwise healthy, will be the almost necessary consequence of the employment of cold water to the extent I have recommended.

I wish to speak the more earnestly on this subject, because I think I have observed, in country practice more especially, many unpleasant consequences to follow from a want of correct ideas in relation to the remedy, which it is the object of this paper to introduce more particularly to the notice of the profession. In the country, as I have before stated, leeches cannot always be had; the application of the scarificator may be frequently inconvenient, and sometimes impossible; and general depletion, unassisted by local treatment, will often prove ineffectual; while in cold water we have a remedy abundantly found throughout the whole of our widely extended country, and possessed of virtues which should long ago have attracted to it much more attention than it has hitherto received. In our cities, also, I am convinced, its employment might be very beneficially extended.

Gangrene, consequent upon violent inflammation, though by no means of such frequent occurrence as formerly, is still met with sufficiently often to call forth all our exertions for its prevention; and I am acquainted with no remedy better calculated to produce such an effect than the one which I have recommended. The present treatment of such cases appears to me to be particularly faulty. A patient, for example, is brought into one of our hospitals with a large lacerated wound of one of the extremities: the surgeon immediately perceives that union by the first intention is, in many cases, impracticable, and that suppuration is the next most desirable termination: to promote which a warm poultice of bread and milk, or some other emollient substances, is immediately applied. It is true that, if the inflammation transcend, as is almost always the case, the degree requisite for the formation of pus, leeches are directed, and the antiphlogistic plan generally is adopted; but in some instances this change of treatment is not made until the inflammation has attained a height which admits of no control, and in others it would have proved inadequate from the commencement; and, even when successful, it is attended with unnecessary loss of time and confinement of the patient. In all such cases, I am fully convinced that the free application of cold water would tend more effectually than any thing else to control inflammatory action, and accelerate the cure.

In fractures, whether simple or compound, the action necessary to reunion is, I believe, very little greater than that of the ordinary healthy action of the part; and I am disposed to think that, in most instances, the process might be much shortened by an attention to this circumstance. A case has been related to me, upon unquestionable authority, of fracture of both bones of the leg, a little above the ankle, followed by reunion in *nine* days. The person had fallen into a well, where he lay nearly an hour before assistance could be rendered him: after being taken out, cold water was applied, as above recommended; and, upon entering the apartment on the morning of the ninth day, the physician in attendance was not a little surprised to find his patient sitting on the side of the bed, with his foot on the floor. Upon examination, the bone, though not entirely consolidated, was found sufficiently so to admit of the abandonment of all artificial support.

Nothing has been farther from my object, in this paper than to depreciate the value of general and local depletion in the treatment of all inflammatory affections: they are, indeed, indispensable. I have only sought to raise from the situation of a humble auxiliary, a remedy which some little experience has taught me is entitled to rank as a principal.

---

### HOSPITAL REPORTS,

